# Linkage analysis of the GAW14 simulated dataset with microsatellite and single-nucleotide polymorphism markers in large pedigrees

**DOI:** 10.1186/1471-2156-6-S1-S14

**Published:** 2005-12-30

**Authors:** Xiaohong (Rose) Yang, Kevin Jacobs, Kimberly F Kerstann, Andrew W Bergen, Alisa M Goldstein, Lynn R Goldin

**Affiliations:** 1 Division of Cancer Epidemiology and Genetics, National Cancer Institute, NIH, DHHS, Bethesda, Maryland, USA; 2 BioInformed LLC, Cleveland, OH, USA

## Abstract

Recent studies have suggested that a high-density single nucleotide polymorphism (SNP) marker set could provide equivalent or even superior information compared with currently used microsatellite (STR) marker sets for gene mapping by linkage. The focus of this study was to compare results obtained from linkage analyses involving extended pedigrees with STR and single-nucleotide polymorphism (SNP) marker sets. We also wanted to compare the performance of current linkage programs in the presence of high marker density and extended pedigree structures. One replicate of the Genetic Analysis Workshop 14 (GAW14) simulated extended pedigrees (*n *= 50) from New York City was analyzed to identify the major gene D2. Four marker sets with varying information content and density on chromosome 3 (STR [7.5 cM]; SNP [3 cM, 1 cM, 0.3 cM]) were analyzed to detect two traits, the original affection status, and a redefined trait more closely correlated with D2. Multipoint parametric and nonparametric linkage analyses (NPL) were performed using programs GENEHUNTER, MERLIN, SIMWALK2, and S.A.G.E. SIBPAL. Our results suggested that the densest SNP map (0.3 cM) had the greatest power to detect linkage for the original trait (genetic heterogeneity), with the highest LOD score/NPL score and mapping precision. However, no significant improvement in linkage signals was observed with the densest SNP map compared with STR or SNP-1 cM maps for the redefined affection status (genetic homogeneity), possibly due to the extremely high information contents for all maps. Finally, our results suggested that each linkage program had limitations in handling the large, complex pedigrees as well as a high-density SNP marker set.

## Background

Previous studies have suggested that a high-density single-nucleotide polymorphism (SNP) marker set could provide equivalent or even superior information compared with currently used microsatellite (STR) marker sets for genome-wide scans by linkage [1-3]. To date, the use of SNP-based linkage mapping has been explored primarily in nuclear families and sib pairs; few studies have evaluated methodological issues involved in SNP linkage using complex or extended pedigrees. This can be challenging because those data frequently overwhelm the computational abilities of the currently available linkage programs to handle simultaneously both the high density of markers and the size of pedigrees. The focus of this study was to evaluate the use of SNP markers for mapping genes in complex pedigrees and to compare the linkage signals to those obtained using STR markers with simulated data from the Genetic Analysis Workshop 14 (GAW14).

## Methods

Parametric and nonparametric linkage analyses (NPL) were used to map the D2 locus with chromosome 3 markers provided in the GAW14 simulated data. Because our goal was to compare the linkage results obtained by using different marker sets and different test statistics, we chose to know the true simulation model before the analyses were performed.

### Replicate and population

Replicate 4 was identified as the largest dataset among the first 10 replicates and thus was chosen for all analyses. Analyses were also conducted using replicate 10 to make certain that our results were not biased due to selection of a non-representative replicate. We selected families from the New York City (NYC) (*n *= 50) cohort because they contained 3 generation pedigrees with at least 4 affected individuals.

### Phenotype

Kofendrerd Personality Disorder (KPD) was modeled as a heterogeneous disease consisting of three phenotypes (P1, P2, and P3) with four genetic loci (D1, D2, D3, and D4) involved. We chose the D2 locus as the major gene to be mapped in this study. The trait variable was analyzed in two ways. The first approach used the original affection status as the disease phenotype. Second, in an attempt to increase the underlying genetic homogeneity, we redefined affection status by classifying individuals who had all four subclinical traits e, f, h, and k as affected. Among these four subclinical traits, e, f, and h involved only D2, and trait k involved D2 and D4, as the major genetic susceptibility loci. Other trait combinations involving loci other than D2 were considered as unaffected.

### Genotype and marker data

D2 was located at the telomeric end of chromosome 3. We analyzed all chromosome 3 STR markers (7-cM average spacing) and original SNPs (3-cM average spacing). In addition, we also "purchased" three 20-marker packets (152, 153, 154) containing 45 telomeric SNPs (B03T3021 to B03T3067) in a 12-cM region on telomeric chromosome 3, with an average spacing of 0.3 cM. To compare the linkage signal with SNP marker sets of different density, we created a 1-cM SNP marker set by only selecting every fourth marker on the dense (0.3 cM) SNP map. All genotype data from founders were removed to decrease the available linkage information content and to more closely resemble realistic situations. Information content of each marker set was measured using the entropy function in MERLIN [4].

### Linkage analysis

All families in the selected replicate were included in the analysis of the original trait. With the redefined affection status, 17 families became uninformative, consisting of either ≤ 1 affected individual within a family or containing only parent-offspring affected pairs. These families were removed when analyzing the redefined affection status and the remaining 33 families were included in all linkage analyses. We performed two-point LOD-score analysis using the MLINK program from the LINKAGE package [5], version FASTLINK 4.1P [6,7], and multipoint parametric linkage analysis using GENEHUNTER 2.1_r5 beta [8], under the assumption of autosomal dominant inheritance of a disease allele with low penetrance (30%) and population frequency of 0.15. These parameter values were obtained from the disease model provided in the true simulation models. We also performed NPL analyses using the programs MERLIN, GENEHUNTER, SIMWALK2 [9], and S.A.G.E. SIBPAL [10]. Evidence for linkage was evaluated with regard to both the magnitude (LOD scores and NPL *p*-values) using all linkage programs and the precision of the peak as determined by the 1-LOD interval from multipoint analyses using GENEHUNTER.

## Results

Results from the different linkage analyses are presented in Table 1 (redefined affection status) and Table 2 (the original affection status), respectively. Because of the large sibships with little missing genotypic data, information content was high for all marker sets. Significant linkage to the redefined trait was successfully detected with all marker sets (STR, SNP-3, 1, 0.3 cM) using all linkage programs. The weakest linkage signal was obtained from analyses using the SNP-3 cM map, which had the lowest information content among all the marker sets. Multipoint parametric HLOD scores obtained from the dense SNP maps (1 cM and 0.3 cM) by GENEHUNTER were much higher compared with those obtained from the STR and SNP-3 cM maps. However, overall results obtained from the STR and dense SNP maps were similar in terms of the magnitude and the precision of the linkage peak, despite the higher information content of the densest SNP-0.3 cM map. Linkage to the original trait, as compared to the redefined trait, was less significant with all marker sets. Under the situation of heterogeneity, the 0.3 cM-map, with the highest information content, was superior, in terms of both magnitude and the precision of the linkage signal, to the other maps.

We obtained similar results with a different replicate (replicate 10), thus our findings were unlikely to be caused by a replicate effect.

## Discussion

In this study, we evaluated the use of SNP markers at different densities in linkage analysis involving large pedigrees and compared the results with those obtained using STR markers. Our results suggested that, for complex pedigrees provided in this simulated dataset, dense SNP marker sets did not provide significantly more information for gene mapping than STR markers at much lower density under the situation of genetic homogeneity. High-density SNPs might detect linkage signals with more precision, that is, with narrower linkage peaks, compared with STRs. However, the difference in 1-LOD intervals obtained from the STR and dense SNP maps (1 cM and 0.3 cM) was not significant (~1 cM) (Table 1). Compared to the SNP-1 cM map, the SNP-0.3 cM map had a higher information content, but it only minimally increased the evidence for linkage and narrowed down the disease gene region. Our data implied that extremely dense SNPs may not necessarily offer great advantage in increasing the power of detecting linkage compared to STRs or SNPs at standard density (1 cM). This is probably because the power for detecting linkage with the redefined affection status was more than adequate even with the less dense SNP map. In fact, information content was high for all three marker sets (STR, SNP-1, SNP-0.3 cM).

To reduce information content, we performed linkage analyses with the same maps using the original affection status, which reflected a situation of genetic heterogeneity. Among the four maps examined, the 0.3-cM map detected linkage to the original trait with the highest significance and precision. Although this was generally consistent with the recent findings from STR-SNP comparisons involving real datasets of nuclear or small extended pedigrees [11-13], the improvement of linkage signals with a dense SNP map observed in our study was less significant compared with those studies. Our finding reflected the near perfect situation of the simulated data in which almost all families had large sibships. Thus, there was little missing genotypic data and phase information could be easily reconstructed. When analyzing real datasets with more extensive missing data, a denser SNP map may be more informative. In fact, the information content of the 3-cM map used in this simulated data set was higher compared with most marker sets from real datasets. It is also likely that STR markers are more effective in capturing genetic correlation among relatives in the complex pedigrees [14].

In linkage studies involving high-density SNPs, one may face the challenge of analytical complexity when mapping genes in large pedigrees. GENEHUNTER and MERLIN, which both use the Lander-Green algorithm, cannot handle a large number of study subjects. GENEHUNTER and MERLIN dropped up to 15 and 9 genotyped individuals from one family in the linkage analyses, respectively. To minimize the problem associated with pedigree size, we also analyzed the data using SIMWALK2, which uses Markov chain Monte Carlo (MCMC) and simulated annealing algorithms in multipoint analyses. However, results from SIMWALK2 yield estimated statistics, in contrast to GENEHUNTER and MERLIN, which provide exact statistics. In addition, it may be difficult to guarantee the adequate convergence of the program; good approximations may require extensive computer processing time, especially when the marker spacing is very dense. In this study, we were unable to obtain a result when analyzing the SNP-0.3 cM marker set because of the failure of convergence. Compared with NPL *p*-values obtained from other linkage programs, *p*-values obtained from SIMWALK2 were conservative. The regression-based SIBPAL program was also used to calculate *p*-values and empirical *p*-values and the results were similar to those obtained from the other statistics, with the exception of a highly significant linkage with the less dense SNPs (3 cM). Finally, although we did not evaluate the impact of linkage disequilibrium (LD) among SNPs on linkage findings due to the limited LD simulated in this region, previous work suggested that the presence of LD among SNPs on a dense SNP map might cause inflated LOD scores [13].

## Conclusion

Extremely dense SNP maps did not provide significant improvement in linkage signals compared with STRs with lower information content when phase information was easily reconstructed (little missing genotypic data, extended pedigree structures, etc.) and when there was genetic homogeneity. Further development and improvement of linkage programs are needed to accommodate the utilization of dense SNP markers in complex pedigrees.

## Abbreviations

GAW: Genetic Analysis Workshop

KPD: Kofendrerd Personality Disorder

MCMC: Markov chain Monte Carlo

NPL: Nonparametric linkage

SNP: Single-nucleotide polymorphism

STR: Short tandem repeat polymorphism

## Authors' contributions

XY performed all linkage analyses and wrote the manuscript, KJ provided data management support and technical consulting, KFK and AWB participated in the design, analysis and result interpretation phases of this study and helped to draft the manuscript, and AMG and LRG provided analysis direction and recommendations at each phase of the analysis.

## Figures and Tables

**Table 1:** Results of linkage analyses of replicate 4 using redefined affection status.

Results from linkage analyses	Type of marker set			
	
	STR-7.5 cM	SNP-3 cM	SNP-1 cM	SNP-0.3 cM
Information content^a^	0.90	0.79	0.87	0.96
Two-point LOD score^b^	3.91	2.22	2.22	3.19
Multipoint Z_max_/NPL *p*-values^c^				
GENEHUNTER				
NPL *p*-value	0.000021	0.0026	3.23e-06	2.16e-07
Multipoint HLOD (α)	3.65 (1.00)	2.45 (0.96)	5.93 (1.00)	6.36 (1.00)
MERLIN				
NPL *p*-value	<0.00001	0.00004	<0.00001	<0.00001
SIMWALK2				
NPL *p*-value	0.0001	0.0066	0.0012	ND^e^
SIBPAL				
*p*-value	5.8 × 10^-7^	9.5 × 10^-8^	4.8 × 10^-6^	7.0 × 10^-7^
1-LOD interval (cM)^d^	5.23	12.55	8.16	7.54

^a^Averaged information content estimated from all markers on STR, SNP-1 cM, SNP-0.3 cM marker sets and the telomeric 50 markers on SNP-3 cM map using the entropy function in MERLIN.

^b^Reflected the highest LOD scores obtained at the telomeric region.

^c^Linkage signal at the telomeric region of chromosome 3 was successfully detected by all marker panels. LOD scores and *p*-values reflected the most significant results for each panel.

^d^Estimated from multipoint LOD-score curves obtained from GENEHUNTER analyses.

^e^Not determined because of the failure in convergence.

**Table 2:** Results of linkage analyses of replicate 4 using original affection status.

Results from linkage analyses>	Type of Marker Set			
	
	STR-7.5 cM	SNP-3 cM	SNP-1 cM	SNP-0.3 cM
Information content^a^	0.84	0.75	0.86	0.96
Two-point LOD score^b^	0.93	0.31	1.97	1.97
Multipoint Z_max_/NPL *p*-values^c^				
GENEHUNTER				
NPL *p*-value	0.0026	0.033	0.0028	0.00084
Multipoint HLOD (α)	1.34 (0.51)	0.49 (0.36)	2.54 (0.70)	2.91 (0.74)
MERLIN				
NPL *p*-value	0.0006	0.005	0.0006	0.0001
SIMWALK2				
NPL *p*-value	0.0016	0.1153	0.0055	ND^e^
SIBPAL				
*p*-value	0.0114	0.0203	0.0198	0.00014
1-LOD interval (cM)^d^	7.85	20.16	12.14	5.16

^a^Averaged information content estimated from all markers on STR, SNP-1 cM, SNP-0.3 cM marker sets and the telomeric 50 markers on SNP-3 cM map using the entropy function in MERLIN.

^b^Reflected the highest LOD scores obtained at the telomeric region.

^c^Linkage signal at the telomeric region of chromosome 3 was successfully detected by all marker panels. LOD scores and *p*-values reflected the most significant results for each panel.

^d^Estimated from multipoint LOD-score curves obtained from GENEHUNTER analyses.

^e^Not determined because of the failure in convergence.
